# Molecular Processes Connecting DNA Methylation Patterns with DNA Methyltransferases and Histone Modifications in Mammalian Genomes

**DOI:** 10.3390/genes9110566

**Published:** 2018-11-21

**Authors:** Albert Jeltsch, Julian Broche, Pavel Bashtrykov

**Affiliations:** Institute of Biochemistry and Technical Biochemistry, Department of Biochemistry, University of Stuttgart, 70569 Stuttgart, Germany; Julian.Broche@ibtb.uni-stuttgart.de (J.B.); Pavel.Bashtrykov@ibtb.uni-stuttgart.de (P.B.)

**Keywords:** DNA methylation, DNA methyltransferase, histone modification, molecular epigenetics

## Abstract

DNA methylation is an essential part of the epigenome chromatin modification network, which also comprises several covalent histone protein post-translational modifications. All these modifications are highly interconnected, because the writers and erasers of one mark, DNA methyltransferases (DNMTs) and ten eleven translocation enzymes (TETs) in the case of DNA methylation, are directly or indirectly targeted and regulated by other marks. Here, we have collected information about the genomic distribution and variability of DNA methylation in human and mouse DNA in different genomic elements. After summarizing the impact of DNA methylation on genome evolution including CpG depletion, we describe the connection of DNA methylation with several important histone post-translational modifications, including methylation of H3K4, H3K9, H3K27, and H3K36, but also with nucleosome remodeling. Moreover, we present the mechanistic features of mammalian DNA methyltransferases and their associated factors that mediate the crosstalk between DNA methylation and chromatin modifications. Finally, we describe recent advances regarding the methylation of non-CpG sites, methylation of adenine residues in human cells and methylation of mitochondrial DNA. At several places, we highlight controversial findings or open questions demanding future experimental work.

## 1. Introduction

In mammals, cytosine residues are methylated at levels between 3.5 and 4.5% in adult tissues depending on the cell type; lower levels are observed in embryonic cell lines and rapidly-dividing cells [1,2]. DNA methylation is a major chromatin regulator and an important part of the epigenome network essential for the development of mammals, which functions in concert with other epigenome modifications, most prominently histone tail modifications [3,4]. Aberrant DNA methylation has several connections to diseases including cancer [5,6], and DNA methylation-changing compounds are in development and clinical use for cancer treatment [7,8].

**Figure 1:**
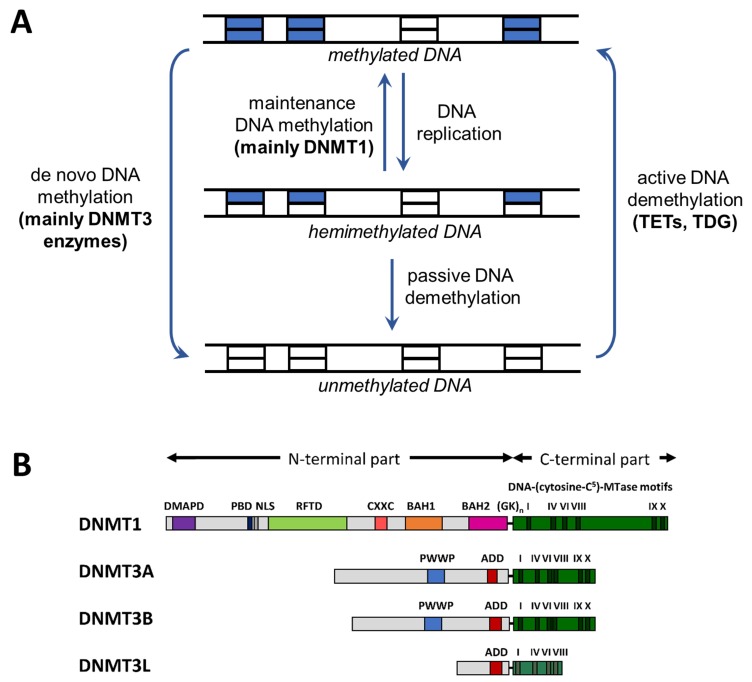
Cycle of DNA methylation and domain structure of DNMTs. (**A**) Cycle of DNA methylation in human cells (adapted from [9]). DNA methylation patterns are generated by de novo methyltransferases and kept through DNA replication by maintenance methylation. DNA methylation can be lost through passive or active demethylation (abbreviations: TET, ten eleven translocation enzyme; TDG, thymine-DNA glycosylase). (**B**) Domain structure of the mammalian DNMTs DNMT1, DNMT3A, and DNMT3B. DNMT3L is a catalytically-inactive member of the DNMT3 family, which has regulatory roles [15]. The human DNMT1, DNMT3A, DNMT3B, and DNMT3L proteins consist of 1616, 912, 853, and 387 amino acid residues, respectively. Abbreviations used: DMAPD, DNA methyltransferase-associated protein 1 interacting domain; PBD, PCNA binding domain; NLS, nuclear localization signal; RFTD, replication foci targeting domain; CXXC, CXXC domain; BAH1 and BAH2, bromo-adjacent homology domains 1 and 2; GK_n_, glycine lysine repeats; PWWP, PWWP domain; ADD, ATRX-DNMT3-DNMT3L domain (reprinted from [15] with permission).

DNA methylation mainly occurs at palindromic CpG sites (28 million sites in the case of the diploid human genome), which are methylated to 70–80%, but cytosines in non-CpG sites are methylated, as well (see below). At CpG sites, the methylation information is present in both DNA strands, meaning that after DNA replication, it can be recovered by a maintenance DNA methyltransferase with high preference for hemimethylated CpG sites, as proposed in the original maintenance DNA methylation model [9] (Figure 1A). Here, we describe DNA methylation patterns in human and mouse DNA in the context of their evolution and compiled information on their correlation with important histone post-translational modifications. Moreover, we describe mechanistic features of mammalian DNA methyltransferases (DNMTs) that contribute to the crosstalk between DNA methylation and chromatin modifications. Finally, we describe recent advances regarding the methylation of non-CpG sites, methylation of adenine residues in human cells, and methylation of mitochondrial DNA. For more detailed reviews on DNA methylation patterns and functions of DNA methylation, refer to [9,10,11,12,13], and for reviews describing the enzymology of DNMTs to [14,15,16]. This review will not focus on DNA methylation in other species like plants, fungi, or arthropods, where many (though not all) of the basic processes are conserved, but additional phenomena are observed. We will also not present the details of DNA methylation recognition and processes involved in DNA demethylation. In these fields, the reader is referred to excellent alternative reviews published recently [17,18,19].

DNA methylation is introduced by a family of enzymes called DNA methyltransferases, which all use S-adenosyl-l-methionine as the methyl group donor (reviews: [14,15,16,20]. In mammals, three active DNMTs are present. DNMT1 is a maintenance methyltransferase with high preference for hemimethylated CpG sites [14,15]. In contrast, the DNMT3A and DNMT3B enzymes do not show preference for hemimethylated target sites, and they are involved in the de novo generation of DNA methylation patterns during germ cell development and the early embryonic phase. All mammalian DNMTs contain a C-terminal catalytic domain, which has structural and sequence homology to prokaryotic DNA-(cytosine C5)-methyltransferases and a larger N-terminal part with different domains involved in targeting and regulation (Figure 1B). DNA demethylation is initiated by the action of the TET family dioxygenases, which catalyze the oxidation of methylcytosine [19].

Despite its overarching elegance, recent data show that the maintenance DNA methylation model cannot fully describe many data, and in fact, all enzymes (DNMT1, DNMT3, and TET enzymes) have roles in DNA methylation after replication and in the de novo generation of DNA methylation and its removal [9]. Therefore, the level of DNA methylation at each cytosine is described by a dynamic equilibrium between gain and loss of methylation [9]. The dynamic nature of DNA methylation patterns has recently been illustrated by showing that the combined knock-out of all TET enzymes leads to hypermethylation of bivalent promoters in human embryonic stem cells (ESC), which was dependent on DNMT3B binding to these sites [21]. Mathematical models have been developed to describe global changes of DNA methylation depending on the expression levels of DNMTs and TETs during serum-to-2i transition of ESCs [22] and gametogenesis [23]. For simulation of local site-specific methylation levels, these models have to be expanded, including the local targeting and preferences of DNMTs and TETs, binding of other proteins, and regulation of DNMTs and TETs, which would usher in a new era of quantitative epigenomics system biology.

## 2. Evolutionary Impact of DNA Methylation

**Figure 2:**
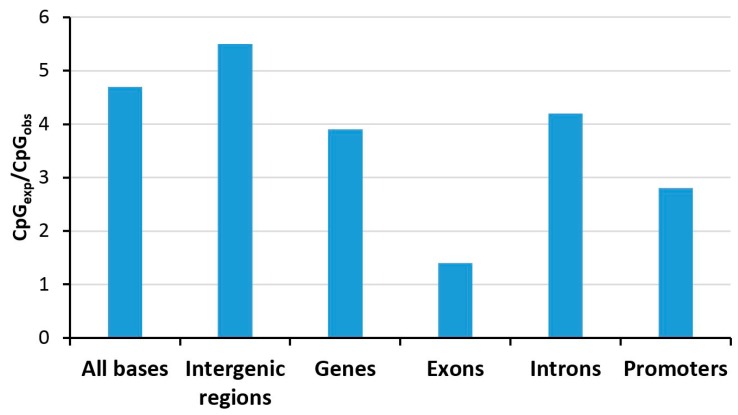
Depletion of CpG dinucleotides in defined genomic elements tabulated for human chromosome 1 [29]. Abbreviations used: CpG_exp_, expected number of CpG sites (based on the nucleotide composition); CpG_obs_, observed number of CpG sites.

Due the presence of the methyl group, 5-methylcytosine (5mC) is more prone to deamination, resulting in thymine-guanine (TG) mismatches occurring in a CpG sequence context. This lesion cannot be repaired via the canonical uracil DNA deglycosylase pathway, which otherwise repairs deamination of cytosine, but it requires specific DNA repair enzymes including MBD4 and thymine DNA glycosylase (TGD) in human cells (review: [24]). However, in spite of the presence of these specialized TG mismatch base excision repair systems, the reversal of 5mC deamination is incomplete, and 5mC is mutagenic, which led to a depletion of the genome from methylated CpG sites over evolutionary times. This is illustrated by the fact that the CpG dinucleotide is currently about 4–5-times less abundant than expected on the basis of the single nucleotide frequencies in human DNA (Figure 2), while GpC sites, which are not methylated, are observed roughly at expected frequencies. However, this CpG depletion did not occur (or it is less pronounced) at certain regions, so-called CpG islands (CGI). Typical definitions for CGIs are an average GC frequency of ≥50% and CpG observed/expected ratio of ≥0.6 in regions of ≥400–500 bps [25]. CGIs occur in the promoters of 70% of all genes, and typically, they are not methylated, explaining the local lack of CpG depletion over evolutionary times and the overall reduced depletion of CpG sites in promoters (see below for more details on the methylation of CGIs). Interestingly, the strong selection pressure on exons apparently has drastically diminished the CpG depletion in these genetic elements (Figure 2).

Recently, another unexpected evolutionary impact of DNA methylation and DNMTs has been discovered by showing that different DNMTs (bacterial M.SssI and mouse DNMT3A catalytic domain) also generate low levels of 3 mC [26]. This modified base represents a DNA damage, which is mutagenic and results in a strong replication block. However, this lesion can be directly repaired by ALKB2 family enzymes in an oxidative process [27,28]. Interestingly, it has been found that ALKB2 enzymes are evolutionarily connected with active DNMTs in many species, suggesting their functional relationship [26].

## 3. Genomic Distribution and Variability of DNA Methylation

**Figure 3:**
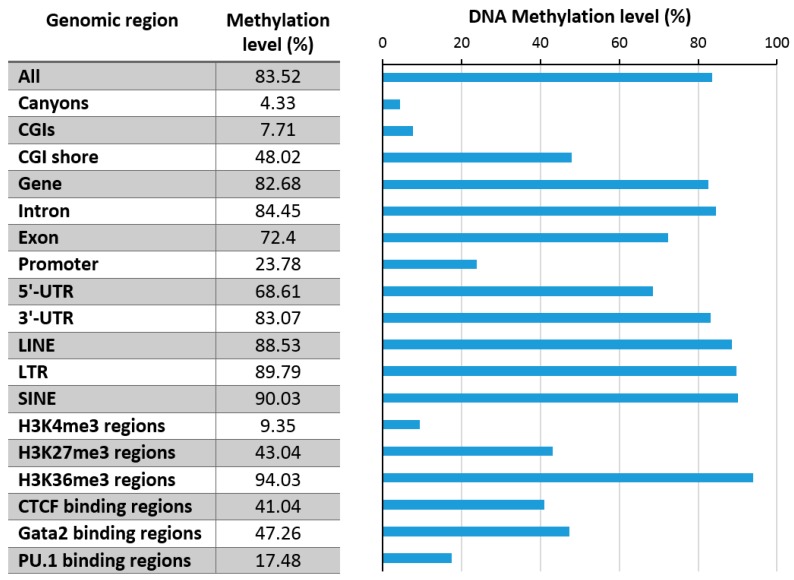
Exemplary DNA methylation levels in mouse hematopoietic stem cells in various genome regions (data taken from [11]). CGI: CpG islands, UTR: untranscribed region, SINE: Short Interspersed Nuclear Elements, LINE: Long Interspersed Nuclear Elements, LTR: Long Terminal Repeat

Early studies have demonstrated that the DNA methylation in human DNA shows a biphasic distribution in which CpG sites are either unmethylated or fully methylated [30,31]. Relatively few sites have intermediate methylation levels, which would point towards a heterogeneous methylation state of the corresponding CpG site in the sample. This heterogeneity can arise from cellular heterogeneity, allelic heterogeneity, cell cycle-dependent heterogeneity or fluctuating levels of methylation at one allele. A recent study showed that heterogeneous methylation largely reflects asynchronous proliferation in normal cells, while cancer cells showed more replication-independent heterogeneity [32]. Numerous datasets revealed that DNA methylation is also unequally distributed among genomic elements (Figure 3), and it is correlated with other chromatin marks, which will be described in the following sections in more detail.

### 3.1. Promoter Methylation

There are about 30,000 CGIs in the human genome, and about 70% of all gene promoters are connected to a CGI. CGIs correspond to only 0.67% of the overall genome, but they nevertheless contain approximately 7% of all CpG sites. Methylation of promoter CGIs in normal cells is usually restricted to stable silencing as in X-chromosome inactivation or imprinted genes, but aberrant CGI methylation is observed in cancer cells. However, non-promoter CGIs can become methylated in human tissues in a tissue-specific manner [33]. Moreover, in cell lines and most cancers, hundreds of CGIs are hypermethylated, and in some cases, a so-called CGI methylator phenotype (CIMP) is observed with thousands of CGIs being hypermethylated [34].

Promoters can be differentiated into high CpG (HCG), intermediate CpG (ICG), and low CpG (LCG) promoters, and early genome-wide DNA methylation analyses showed a clear anticorrelation of CpG density and DNA methylation [30,31,35]. Therefore, HCG and LCG usually show low and high DNA methylation, while ICGs show the most variability. These include regions flanking CGIs, so-called CGI shores, which have moderately-elevated CpG frequencies. An analysis of DNA methylation data of 30 human cell and tissue types revealed that methylation of about 20% of the CpG sites are dynamically regulated [36], but this number is likely to increase further as more datasets are incorporated. Most variances in DNA methylation were indeed observed in ICGs and CGI shores.

As mentioned above, CGIs are generally protected from DNA methylation. Different mechanisms appear to be involved in this process: (1) It has been shown that this protection is sequence dependent, suggesting that bound transcription factors (TFs) prevent methylation [37]. Mechanistically, TFs can physically preclude access of DNMTs [38]. Moreover, bound TFs can lead to the deposition of activating marks, particularly H3K4me2/3, which prevents binding of DNMT3 enzymes (see below). The cell type-specific binding of TFs can also explain the strong overrepresentation of regulatory elements in regions that show differential methylation during development [36]. In agreement with this general model, it has been shown that allele-specific methylation often occurs at regulatory sites where single-nucleotide polymorphisms between alleles cause differential binding of TFs [39,40]. (2) CGIs can be protected from methylation by association with TET enzymes. Studies in murine ESCs [41], as well as human embryonic kidney cells (HEK293T) [42] have shown that TET1 strongly binds to CpG-rich DNA associated with high-CpG-density gene promoters and exons, and its density is positively correlated with H3K4me3 in promoter regions. Furthermore, TET2 and TET3 were shown to bind to CpG islands and promoter regions. (3) CXXC domain-containing readers of unmethylated DNA preferentially bind at CGIs, and by this, they recruit other chromatin factors to these regions, which helps to keep them unmethylated [43]. These domains are found in numerous chromatin factors, and they appear to be involved in their targeting to unmethylated CGIs [44], including the KDM2A and KDM2B H3K36-specific lysine demethylases [45], the KMT2A and KMT2B H3K4-specific protein lysine methyltransferases (PKMTs) [46], as well as TET1 and TET3 [47]. This model has recently been further validated experimentally by showing that CXXC domains recruit TET enzymes to unmethylated CpG-rich CGIs, leading to the DNA demethylation and protection of these regions from de novo methylation [21].

Methylated CGIs recruit classical MBD family readers of DNA methylation including MeCP2, MBD1, or MBD2, which establish strong repression of gene expression by containing transcriptional repression domains and forming complexes with other silencing factors including lysine deacetylases, H3K9 methyltransferases (SUV39H1) and chromatin remodelers (review: [18]). In addition, the SETDB1 H3K9 methyltransferase, which has a prominent role in the silencing of repeats and retrotransposons, also contains an MBD domain. However, it has become clear that MBD proteins are not exclusively involved in gene silencing. For example, MeCP2 can function as a gene activator and repressor, as shown by gene expression studies in brain regions where most target genes were found to be upregulated by MeCP2 [48,49,50]. In agreement with these findings, MeCP2 was shown to interact with DNMT3A and function as an inhibitor or stimulator of DNMT3A activity, depending on the chromatin context [51].

### 3.2. Enhancer Methylation and Influence of DNA Methylation on TF Binding

Enhancers functionally resemble promoters by containing binding sites for TFs, but they show depletion of CpG sites roughly corresponding to the average genome. Recently, tissue-specific DNA methylation changes have been detected mainly in enhancers during early development and postnatally, which were triggered by de novo methylation and demethylation [52]. Methylation of TF binding sites in CpG-poor promoters or in enhancers can have variable effects on gene expression, depending on its influence on DNA binding of the TF (repelling or enhancing) and the specific role of the TF (activating or repressing). DNA methylation can prevent the binding of several TFs [53,54]. For example, the CTCF protein, composed of a linear array of 11 zinc finger domains, binds to DNA in a methylation-dependent manner, but methylation prevents binding only at some binding sites [55]. This was recently explained by structural data showing that DNA methylation only affects binding if it occurs at one particular position within the consensus binding site [56]. However, DNA methylation can also promote DNA binding of TFs [53,54]. For example, there is a group of C2H2 zinc finger proteins that bind methylated DNA in a sequence-dependent manner [57,58]. One of them is Kaiso, which interacts with the N-CoR repression complex. Another member of this group is ZFP57, a KRAB zinc finger, which recruits the KAP1 corepressor to methylated imprinting control regions. At the cellular level, the modulation of the DNA binding of chromatin-organizing proteins like CTCF and cohesins by DNA methylation has been connected to altered genome structure and cancer [59]. So-called pioneering TFs were shown to bind to heterochromatic and methylated enhancers, leading to their activation, which is accompanied by a slow loss of DNA methylation [60]. These processes are essential steps in the differentiation of cell lineages. Similarly, KLF4 binding to methylated target sites was shown to mediate gene activation [61].

In agreement with an activating role of DNA methylation at some enhancers, it has been shown that H3K27 acetylation and DNA methylation can co-exist at enhancers. These bivalent enhancers were shown to lose acetylation after removal of DNA methylation, implying that DNA methylation was needed for the maintenance of the acetylation mark [62]. Recently, the binding of DNMT3A and DNMT3B to enhancers was studied, showing that both proteins associate with most active enhancers in epidermal stem cells [63]. This binding was dependent on H3K36me3, suggesting that it is mediated by the PWWP domain binding to this mark [15,16]. Interestingly, both DNMT3 proteins differ in their effects on enhancer DNA modification. DNMT3B was shown to be involved in enhancer body methylation, while DNMT3A was shown to cooperate with TET2, promoting enhancer DNA hydroxymethylation. Interestingly, both DNMT3A and DNMT3B are required for enhancer activity and enhancer RNA production, illustrating the dual regulatory potential of DNA methylation for gene repression and activation.

### 3.3. Repeat Methylation

DNA methylation levels of repeats are generally high, in line with the classical function of DNA methylation to repress the transcriptional activity of repeats and thereby protect genome integrity. There are four classes of highly abundant repeat elements in the human genome: Short Interspersed Nuclear Elements (SINEs), Long Interspersed Nuclear Elements (LINEs), Long Terminal Repeats (LTRs), and DNA transposons (Table 1). All of them show depletion of CpG sites. DNA methylation at repeats varies through development, and ES cells often show reduced repeat methylation levels. In the case of SINE elements, it has been observed that their methylation in ES cells reflects the effect of CpG density on DNA methylation levels in the general genome. CpG-rich SINE elements, which are relatively rare, tend to show low methylation, while CpG-poor elements, which are more abundant, show high methylation [30]. Recently, the repressive role of DNA methylation on repeats has been experimentally documented by showing that treatment of cells with DNMT inhibitors leads to the derepression of LTRs [64,65].

Targeting of repetitive DNA in part depends on SETDB1 (also known as ESET or KMT1E), a histone H3K9 lysine methyltransferase that generates H3K9me3 in euchromatic regions [67]. It forms a complex with KAP-1 (also called TRIM28) that can further interact with KRAB zinc finger proteins, which are involved in the recognition and silencing of repeats and transposons [68,69]. Moreover, it associates with additional silencing factors including DNMT3A [70]. The KAP-1/SETDB1 complex is required for silencing of LTR retroviruses, and it has also been connected to H3K9 methylation of LINE elements (see the references provided in [67]). Targeting of repetitive DNA in the mammalian germline is also dependent on the piRNA pathway [71], but the details of this process are not known.

### 3.4. DNA Methylation Canyons

Genome-wide DNA methylation analyses unexpectedly revealed the existence of a new class of large hypomethylated regions, which were called canyons or DNA methylation valleys [72,73]. These regions typically span up to 1 MB and contain several CGIs interrupted by DNA with lower CpG content. DNA methylation canyons are conserved among cell types and species and uniquely enriched for TF binding sites and developmental regulatory genes. Canyon borders were shown to be marked by 5-hydroxymethyl cytosine (5hmC), and they become eroded in the absence of DNMT3A [73], suggesting that the edges of these canyons represent regions where active DNA methylation, hydroxymethylation, and demethylation are in a dynamic steady-state.

## 4. Relation of DNA Methylation and Chromatin Marks

### 4.1. H3K4me3

**Figure 4:**
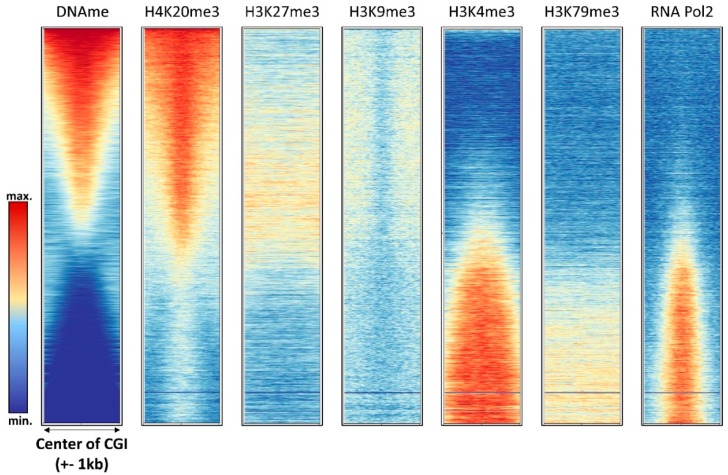
Compilation of DNA methylation density with the density of other chromatin marks in annotated CGIs in HEK293 cells (see Supplementary Text 1 for the data sources).

Early genome-wide DNA methylation analyses revealed one of the most striking features of DNA methylation patterns, that DNA methylation is strongly anticorrelated with H3K4me2/3 [30,31], a finding that has been reproduced in several follow-up studies (Figure 3 and Figure 4). H3K4me3 marks active promoters with high occupancy of RNA polymerase II, also showing elevated levels of H3K79me3 (Figure 4). Low DNA methylation of these regions is in agreement with the fact that the ATRX-DNMT3-DNMT3L (ADD) domains of DNMT3A, DNMT3B, and DNMT3L cannot bind to the H3 tail di- or tri-methylated at H3K4 [15,16]. Moreover, the DNMT3 enzymes require binding of the K4 unmodified tail to their ADD domain to activate the catalytic center, such that DNMT3 enzymes aberrantly bound at H3K4me2/3 regions would remain catalytically inactive.

By this mechanism, H3K4 methylation in active promoters protects these regions from DNA methylation. Mechanistically, the ADD binding of DNMT3 proteins is also disrupted for example by acetylation of K4 or phosphorylation of T3 and T6 [74]. The functional role of the readout of these chromatin marks has been studied experimentally by designing of DNMT3A variants with mutated ADD domains that were no longer sensitive towards K4 methylation or T6 phosphorylation [75]. Expression of these DNMT3A mutants in cells perturbed the differentiation program of ESC, and it led to chromosomal instability. The prominent role of the ADD domain in the targeting of DNMT3 enzymes has recently also been confirmed at enhancers of pluripotency genes, where it was shown that LSD1-dependent demethylation was necessary for DNMTA binding, which led to enhancer methylation [76].

### 4.2. H3K36me3

**Figure 5:**
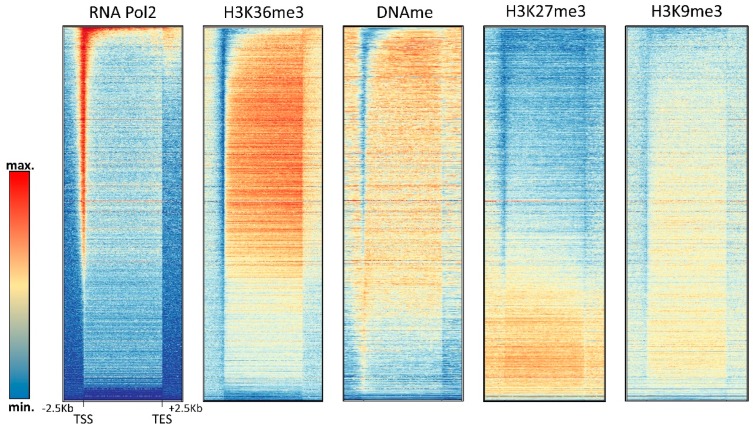
Compilation of DNA methylation density and the density of other chromatin marks in gene bodies in HEK293 cells (see Supplementary Text 1 for the data sources).

H3K36me3 accumulates in the bodies of expressed genes where it is introduced by the SETD2 PKMT, which is recruited by the RNAPII phosphorylated at Ser2 and Ser5 in the C-terminal tail [77,78]. DNA methylation in gene bodies is also particularly high [79], which is in agreement with the binding of the DNMT3A and DNMT3B PWWP domains to the H3K36 methylation mark [15,16]. Within gene bodies, H3K36me3 and DNA methylation are correlated with gene expression, while H3K27me3 and H3K9me3 are anticorrelated with expression (Figure 3 and Figure 5). Using a DNMT3B knock-out mouse ES cell line, it was shown that intragenic DNA methylation is deposited by DNMT3B [80], while the potential role of DNMT3A in this process has remained unclear. Gene body DNA methylation by DNMT3B was shown to be dependent on SETD2-deposited H3K36me3 as expected from the PWWP domain binding this mark [81]. Intragenic DNA methylation has several functions including the regulation of alternative promoters [82] and alternative splicing [83], as well as prevention of intragenic transcription initiation [81], which are in line with the general functions of gene body H3K36 methylation [77]. It is currently unclear if H3K36me3 binding only has a recruiting function for DNMT3 enzymes or if it also regulates their activity. Strikingly, some studies provided evidence that the gene body methylation even has a direct stimulatory role on gene expression by unknown mechanisms [84,85]. Another interesting question is the slightly lower DNA methylation in exons when compared to introns that so far has escaped mechanistic and functional explanation. One possible explanation for this observation could be that exons are more CpG-rich than introns. 

### 4.3. H3K27me3

The relationship of DNA methylation with the Polycomb H3K27me3 mark is complex and ambivalent, because different studies have provided evidence that both marks can either act together or antagonistically. The complex interplay of DNA methylation and H3K27me3 at CGIs is illustrated in Figure 4 showing that highly methylated CGIs in HEK293 cells are depleted from H3K27me3, but CGIs with medium methylation levels show a slight enrichment of H3K27me3. In gene bodies, H3K27me3 seems to be anticorrelated with DNA methylation (Figure 5).

Early studies showed an interaction of DNMTs and PRC2 (the PKMT complex that generates H3K27me3) [86] and demonstrated that H3K27me3 marked genes are targets for aberrant DNA methylation in cancer cells and cell lines [31,87,88]. Later, genome-wide studies in ES cells with knock-out of all DNMTs that are lacking DNA methylation revealed a reduction in localized H3K27me3 peaks, in agreement with the model of a synergistic function of DNA methylation and H3K27me3 [89]. A synergistic function of DNA methylation and H3K27me3 is also supported by the fact that both are enriched at the inactive X-chromosome, where they lead to its transcriptional silencing (review: [90]), and by the recent report that PRC2 preferentially binds CG-rich and CpG methylated DNA in vitro [91].

On the other side, a combined chromatin immunoprecipitation (ChIP)-bisulfite study revealed an antagonism of both marks at CpG islands [89]. In line with this result, other studies found that PRC2 binds to unmethylated, but not to methylated genomic regions, and disruption of DNA methylation leads to the appearance of H3K27me3 at the previously methylated CGIs (review: [92]). The global antagonism of these modifications was also confirmed by showing that DNA methylation valleys contain broad regions of H3K27me3 [72]. It was found that binding of the Polycomb protein, which is part of the PRC1 complex, indeed promotes the hypomethylation of DNA methylation valleys, likely by the regulation of TET enzymes [93].

### 4.4. H3K9me3

Historically, H3K9me3 is tightly connected with DNA methylation, because the *Neurospora crassa* Dim5 enzyme, one of the first-discovered H3K9-specific PKMTs [94], was found in a screen for mutations with reduced DNA methylation. In mammals, both marks are known to be enriched in heterochromatin, but their connection is not yet fully understood. Knock-out of SUV39H1 and SUV39H2 in ESCs has been shown to cause reduced DNA methylation at major satellite repeats, but not at other different repeat elements [95]. Early studies have shown targeting of DNMT3B to heterochromatic sites by HP1 alpha, an H3K9me3 reader [96]. However, the lack of reduction of CpA methylation (which can only be deposited by DNMT3 enzymes) in SUV39 double-knock-out cells [95] suggested that the connection of H3K9me3 and DNA methylation is rather mediated by DNMT1. One candidate for this function is UHRF1 [97], which is an essential factor for DNA methylation in mammals [15,16]. It was discovered in 2007 that UHFR1 co-localizes with DNMT1 and PCNA at replicating heterochromatic regions during mid- to late S-phase, and the association of DNMT1 with chromatin was lost in UHFR1 knock-out (KO) cells [98,99]. UHFR1 KO is embryonically lethal in mice, and UHRF1-deficient embryos showed strongly reduced levels of genome-wide DNA methylation, indicating that UHRF1 has an essential role in the maintenance of DNA methylation. These observations led to a model that UHFR1 recruits DNMT1 to replicated hemimethylated DNA to facilitate its efficient re-methylation. Since this impressive discovery, the structural, mechanistic, and functional details of the DNMT1-UHRF1 interaction have been a subject of very intense investigation. Like DNMT1, UHRF1 is a large multidomain protein [15]. UHRF1 stimulates the catalytic activity of DNMT1 by an interaction with the DNMT1 RFT domain, which opens the auto-inhibited conformation [100,101].

UHRF1 binds to hemimethylated DNA with its SET- and RING-associated (SRA) domain, and its tandem Tudor domain (TTD) and plant homeodomain (PHD) bind H3-tails containing H3K9me3 and unmodified H3R2 in a cooperative reaction [102,103]. H3K9me3 binding of UHRF1 was required for the localization of UHRF1 to heterochromatin and for maintenance of DNA methylation, since a mutation in TTD, which prevents binding of UHRF1 to H3K9me3, abolished both functions [97,104]. Similarly, disruption of H3R2 binding by the PHD domain abolished DNA methylation by DNMT1 in cells [83]. The reduction of H3K9me2 and UHRF1 observed during global demethylation in the serum-to-2i transition in ESCs was also suggestive of a connection of both processes [22]. However, recently, knock-in of a UHRF1 gene with the mutated H3K9me3 binding site into *Uhfr1* deletion cells led to an almost complete recovery of DNA methylation, suggesting that the H3K9me3 binding site of UHRF1 alone is not essential for DNA methylation [105]. UHRF1 binding also mediates the crosstalk of DNA methylation with asymmetrically-dimethylated H3R2, which is introduced by PRMT6. PRMT6 functions as a negative regulator of DNA methylation, because the H3R2 methylation interferes with UHRF1 binding [106]. By this mechanism, overexpression of PRMT6 in cancer cells could be connected to the global DNA hypomethylation often observed in these cells.

Interestingly, elevated DNA methylation is apparently not accompanied by increased H3K9me3 in promoter CGIs; however, promoter CGI methylation is strongly correlated with H4K20me3 (Figure 4). H4K20me3 is another heterochromatic chromatin modification, deposited by the SUV420H1 and SUV420H2 PKMTs [107]. Its role at promoters and the molecular mechanism of its connection with DNA methylation at these sites has not yet been well investigated. In gene bodies, DNA methylation and H3K9me3 are anticorrelated (Figure 5).

### 4.5. Chromatin Remodeling and DNA Methylation

Historically, chromatin remodeling has been very tightly connected to DNA methylation, because in an *Arabidopsis thaliana* screen for mutants with lost DNA methylation, the first discovered mutation did not affect a DNA methyltransferase, but the *Ddm1* gene, a putative chromatin remodeler. The mammalian Ddm1 homolog is called HELLS (previously also LSH), and it was shown to be essential for DNA methylation [108,109]. HELLS is an SNF2 ATPase protein and putative chromatin remodeler, and its ATPase activity was indeed shown to be necessary for chromatin binding and stimulation of DNA methylation [110,111]. The close connection of DNA methylation and chromatin remodeling can be understood on the basis of the structures of DNMT1 [112] and DNMT3A [113] in complex with DNA, because both complexes show that DNMT binding would not be possible to nucleosomal DNA. This conclusion is in agreement with experimental data showing that DNA bound to nucleosomes is not efficiently methylated by DNMTs [114,115,116,117]. Along the same lines, it was demonstrated that nucleosomal DNA can only be methylated by DNMT1 in the presence of chromatin remodelers [118].

Immunodeficiency-centromeric instability-facial anomalies syndrome (ICF) is a hereditary disease characterized by reduced DNA methylation of pericentromeric heterochromatic repeats mainly in chromosomes 1, 9, and 16, which was initially connected to loss or reduction of function mutations in the DNMT3B DNA methyltransferase [119,120]. Recently, it was shown that in addition to DNMT3B mutations, also mutations in HELLS and the ZBTB24 and CDCA7 Zinc finger proteins can cause ICF [121,122]. ZBTB24 has been shown to promote CDCA7 transcription [123], and the complex of HELLS and CDCA7 recently has been shown to have chromatin remodeling activity [124]. These data suggest that the remodeling activity of HELLS/CDCA7 indeed could be necessary for DNMT3B methylation of the pericentromeric heterochromatic repeats.

## 5. Additional DNA Methylation Events

### 5.1. Non-CpG Methylation: Enzymes, Patterns, and Role

Despite clear evidence for the existence of non-CpG methylation in human DNA for a long time, its presence has been documented only during recent years. The reason for this is that it generally occurs at low levels in mammals, which makes it technically difficult to discriminate true non-CpG methylation from incomplete conversion, a regular artifact in bisulfite DNA methylation analysis. Biochemical studies showed that non-CpG methylation can be introduced by DNMT3 enzymes, which have a relaxed specificity [14]. However, DNMT1, the most active and important DNMT in mammals, shows an exquisite specificity and is unable to methylate non-CpG sites [125]. Therefore, while CpG methylation is efficiently duplicated after each cell division, this is not true for non-CpG methylation which must be generated de novo after each DNA replication. Interestingly, this is also true for CHG sites (where H stands for A, T or C), although they are palindromic and contain the methylation information in both DNA strands. In spite of this, DNA methylation at these sites is not “directly maintained”, because in mammals there is no DNMT with specificity for hemimethylated CHG sites. Methylation analyses in DNMT KO cell lines clearly demonstrated that DNMT3 enzymes are indeed responsible for non-CpG methylation [95]. Consequently, non-CpG methylation is mainly found in tissues with high expression of DNMT3 enzymes like embryonic tissues and neurons, where the lack of cell division also favors the stable presence of non-CpG methylation. Non-CpG methylation is observed at levels >2% of sites in neurons and at >1% in frontal cortex and embryonic cells [126]. In the genome, it is enriched in gene bodies and transposons. Interestingly, non-CpG methylation mainly occurs in a CAG context in ES cells and in early development, while in differentiated neurons, mainly CAC methylation is observed [126]. Studies in mouse DNMT KO ES cells after re-expression of DNMT3A or DNMT3B revealed that DNMT3A preferred introduction of CAC methylation, while DNMT3B was more active in CAG methylation [127], which is in agreement with the general observation that DNMT3B has a role in early development and that DNMT3A is highly expressed in brain tissues.

In the brain, CpG and non-CpG (mainly CA) methylation are observed in the gene bodies of long MeCP2 repressed genes [128]. Disruption of CA methylation by conditional DNMT3A knock-out revealed that CA methylation is critical for binding of MeCP2 and repression of long genes [129]. In another study, binding of MeCP2 to non-CpG methylated DNA was found to regulate gene expression in both directions in adult mouse brain [130]. A recent study has investigated the distribution of non-CpG methylation on the active and inactive X-chromosome in murine frontal cortex cells showing that non-CpG methylation was enriched in the active X-chromosome and one transcriptionally-active region of the inactive X-chromosome [131], similarly as shown for gene body methylation on the active X-chromosome previously [35]. While this distribution is easily explained by the transcription-dependent targeting of DNMT3 enzymes via H3K36me3 binding of the PWWP domains, it raises interesting new questions regarding the role and function of non-CpG methylation in mammals.

### 5.2. Rare Methylation Events: Controversies about 6mA and Mitochondrial DNA Methylation

In bacteria, two additional types of naturally-methylated nucleobases occur in DNA, namely 6-methyladenine (6mA) and 4-methylcytosine. For decades, it has been discussed whether particularly the 6mA modification also exists in the DNA of high eukaryotes [132]. With the development of more sensitive liquid-chromatography coupled mass spectrometry (LC-MS) detection systems, several papers recently reported the presence of 6mA in higher eukaryotes including *C. elegans* (0.013–0.39% mdA/dA = 130–3900 ppm, depending on growth conditions) [133], *Drosophila* (10–70 ppm) [134], *Xenopus* (0.9 ppm) [135], and mouse ES cells and tissues (6–7 ppm) [123]. In a more recent paper based on antibody detection, mass spectrometry and single-molecule real-time sequencing reported 6mA levels of 510 ppm [136]. Clearly, even the highest estimates of 6mA in human DNA are below 10^6^ modified adenine residues per diploid genome, indicating that it is a rare modification. The presence of 6mA in mouse DNA has recently been reexamined using highly sophisticated quantitative LC-MS methods [137]. These authors did not find evidence for 6mA in mouse ES cells and tissues with a detection limit of 0.35 ppm. They also reported 6mA contaminations in commercial enzyme preparations and highlighted the possibility of 6mA contamination via bacterial DNA (from skin or gut) or after ingestion of bacterial DNA and incorporation of 6mA into the DNA through the salvage pathway. More carefully-controlled experiments and cross-lab validations will be necessary to clarify this question.

A similar controversy exists about mitochondrial DNA methylation, where papers presented evidence in favor of its existence (mostly based on bisulfite conversion coupled with sequencing) [138]. Mitochondrial DNA methylation had been connected with a mitochondrial isoform of DNMT1 [139], which is in apparent disagreement with another paper reporting that mitochondrial DNA methylation occurs at GpC (not CpG) sites [140], because DNMT1 is a strict CpG methyltransferase. Different recent bisulfite conversion studies coupled with deep sequencing provided evidence that most mitochondrial DNA methylation signals may be caused by technical artifacts of the bisulfite sequencing technology [141,142]. These controversies illustrate that particularly in the cases of low to very low methylation levels, powerful controls must be included in any analysis, and the biological relevance of potential methylation events must be rigorously assessed.

## 6. Outlook

DNA methylation is an essential part of the chromatin modification network, also comprising several covalent histone protein post-translational modifications. All these modifications are highly interconnected, because the writers and erasers of one mark (DNMTs and TETs in the case of DNA methylation) are directly or indirectly targeted and regulated by other marks. Our understanding of the properties of this epigenome network are still immature, and many more experiments investigating individual functional connections between the chromatin marks, but also the global effects will be needed for a detailed quantitative description of this network. In the specific case of DNA methylation, we need to understand better how epigenome marks and chromatin structure target and regulate DNMTs and TET enzymes. A better understanding of the functional connections between different epigenome marks will greatly improve our understanding of developmental processes and finally also propel our abilities in epigenome editing, a bioengineering approach aiming at the durable editing of individual chromatin marks like DNA methylation specifically at defined genome loci, which has many promising applications both in basic research and in molecular medicine [143,144].

## Figures and Tables

**Table 1:** Properties and DNA methylation levels of different repeat types in various cell types. Data taken from [66]. The ranges of methylation levels represent the 25th and 75th percentiles. H1 is a human ES cell line; IMR90 are human fetal fibroblasts. Mouse hematopoietic stem cell (mHSC) data were taken from [11].

Repeat Type	Total Number	Mean Length (bps)	Mean GC Content	Mean CpG_exp/obs_	DNA Methylation
SINE	1,426,563	244	0.50	2.98	H1: 81.4–90.2
					IMR90: 62.8–90.9
					mHSC: 90.0
LINE	947,779	578	0.38	4.01	H1: 82.6–90.7
					IMR90: 41.9–88.4
					mHSC: 88.53
LTR	530,763	443	0.44	4.84	H1: 81.4–90.7
					IMR90: 37.2–83.7
					mHSC: 89.79
DNA Transposon	273,586	272	0.40	3.63	H1: 83.7–91.9
					IMR90: 45.4–90.7

## Supplementary Materials

The following documents are available online at http://www.mdpi.com/2073-4425/9/11/566/s1. Supplemental text 1: Sources of the datasets shown in Figure 4.

## References

[1] Globisch D., Münzel M., Müller M., Michalakis S., Wagner M., Koch S., Brückl T., Biel M., Carell T. (2010). Tissue distribution of 5-hydroxymethylcytosine and search for active demethylation intermediates. PLoS ONE.

[2] Lisanti S., Omar W.A., Tomaszewski B., De Prins S., Jacobs G., Koppen G., Mathers J.C., Langie S.A. (2013). Comparison of methods for quantification of global DNA methylation in human cells and tissues. PLoS ONE.

[3] Allis C.D., Jenuwein T. (2016). The molecular hallmarks of epigenetic control. Nat. Rev. Genet..

[4] Noh K.M., Allis C.D., Li H. (2018). Reading between the lines: “ADD”-ing histone and DNA methylation marks toward a new epigenetic “Sum”. ACS Chem. Biol..

[5] Baylin S.B., Jones P.A. (2011). A decade of exploring the cancer epigenome—Biological and translational implications. Nat. Rev. Cancer.

[6] Bergman Y., Cedar H. (2013). DNA methylation dynamics in health and disease. Nat. Struct. Mol. Biol..

[7] Erdmann A., Halby L., Fahy J., Arimondo P.B. (2015). Targeting DNA methylation with small molecules: What’s next?. J. Med. Chem..

[8] Mirfattah B., Herring J., Tang H., Zhang K. (2017). Probes and targets of DNA methylation and demethylation in drug development. Curr. Top. Med. Chem..

[9] Jeltsch A., Jurkowska R.Z. (2014). New concepts in DNA methylation. Trends Biochem. Sci..

[10] Jones P.A. (2012). Functions of DNA methylation: Islands, start sites, gene bodies and beyond. Nat. Rev. Genet..

[11] Jeong M., Goodell M.A. (2014). New answers to old questions from genome-wide maps of DNA methylation in hematopoietic cells. Exp. Hematol..

[12] Schubeler D. (2015). Function and information content of DNA methylation. Nature.

[13] Ye F., Kong X., Zhang H., Liu Y., Shao Z., Jin J., Cai Y., Zhang R., Li L., Zhang Y.W. (2018). Biochemical studies and molecular dynamic simulations reveal the molecular basis of conformational changes in DNA methyltransferase-1. ACS Chem. Biol..

[14] Jurkowska R.Z., Jurkowski T.P., Jeltsch A. (2011). Structure and function of mammalian DNA methyltransferases. Chembiochem.

[15] Jeltsch A., Jurkowska R.Z. (2016). Allosteric control of mammalian DNA methyltransferases—A new regulatory paradigm. Nucleic Acids Res..

[16] Gowher H., Jeltsch A. (2018). Mammalian DNA methyltransferases: New discoveries and open questions. Biochem. Soc. Trans..

[17] Law J.A., Jacobsen S.E. (2010). Establishing, maintaining and modifying DNA methylation patterns in plants and animals. Nat. Rev. Genet..

[18] Du J., Johnson L.M., Jacobsen S.E., Patel D.J. (2015). DNA methylation pathways and their crosstalk with histone methylation. Nat. Rev. Mol. Cell Biol..

[19] Wu X., Zhang Y. (2017). TET-mediated active DNA demethylation: Mechanism, function and beyond. Nat. Rev. Genet..

[20] Jeltsch A. (2002). Beyond Watson and Crick: DNA methylation and molecular enzymology of DNA methyltransferases. Chembiochem.

[21] Verma N., Pan H., Doré L.C., Shukla A., Li Q.V., Pelham-Webb B., Teijeiro V., González F., Krivtsov A., Chang C.J. (2018). TET proteins safeguard bivalent promoters from de novo methylation in human embryonic stem cells. Nat. Genet..

[22] Von Meyenn F., Iurlaro M., Habibi E., Liu N.Q., Salehzadeh-Yazdi A., Santos F., Petrini E., Milagre I., Yu M., Xie Z. (2016). Impairment of DNA methylation maintenance is the main cause of global demethylation in naive embryonic stem cells. Mol. Cell.

[23] Gahurova L., Tomizawa S.I., Smallwood S.A., Stewart-Morgan K.R., Saadeh H., Kim J., Andrews S.R., Chen T., Kelsey G. (2017). Transcription and chromatin determinants of de novo DNA methylation timing in oocytes. Epigenetics Chromatin.

[24] Bellacosa A., Drohat A.C. (2015). Role of base excision repair in maintaining the genetic and epigenetic integrity of CpG sites. DNA Repair (Amst).

[25] Illingworth R.S., Bird A.P. (2009). CpG islands—‘A rough guide’. FEBS Lett..

[26] Rosic S., Amouroux R., Requena C.E., Gomes A., Emperle M., Beltran T., Rane J.K., Linnett S., Selkirk M.E., Schiffer P.H. (2018). Evolutionary analysis indicates that DNA alkylation damage is a byproduct of cytosine DNA methyltransferase activity. Nat. Genet..

[27] Shen L., Song C.X., He C., Zhang Y. (2014). Mechanism and function of oxidative reversal of DNA and RNA methylation. Annu. Rev. Biochem..

[28] Fedeles B.I., Singh V., Delaney J.C., Li D., Essigmann J.M. (2015). The AlkB Family of Fe(II)/α-ketoglutarate-dependent dioxygenases: Repairing nucleic acid alkylation damage and beyond. J. Biol. Chem..

[29] Wojciechowski M., Czapinska H., Bochtler M. (2013). CpG underrepresentation and the bacterial CpG-specific DNA methyltransferase M.MpeI. Proc. Natl. Acad. Sci. USA.

[30] Meissner A., Mikkelsen T.S., Gu H., Wernig M., Hanna J., Sivachenko A., Zhang X., Bernstein B.E., Nusbaum C., Jaffe D.B. (2008). Genome-scale DNA methylation maps of pluripotent and differentiated cells. Nature.

[31] Zhang Y., Rohde C., Tierling S., Jurkowski T.P., Bock C., Santacruz D., Ragozin S., Reinhardt R., Groth M., Walter J. (2009). DNA methylation analysis of chromosome 21 gene promoters at single base pair and single allele resolution. PLoS Genet..

[32] Charlton J., Downing T.L., Smith Z.D., Gu H., Clement K., Pop R., Akopian V., Klages S., Santos D.P., Tsankov A.M. (2018). Global delay in nascent strand DNA methylation. Nat. Struct. Mol. Biol..

[33] Lokk K., Modhukur V., Rajashekar B., Märtens K., Mägi R., Kolde R., Koltšina M., Nilsson T.K., Vilo J., Salumets A. (2014). DNA methylome profiling of human tissues identifies global and tissue-specific methylation patterns. Genome Biol..

[34] Pfeifer G.P. (2018). Defining driver DNA methylation changes in human cancer. Int. J. Mol. Sci..

[35] Weber M., Hellmann I., Stadler M.B., Ramos L., Pääbo S., Rebhan M., Schübeler D. (2007). Distribution, silencing potential and evolutionary impact of promoter DNA methylation in the human genome. Nat. Genet..

[36] Ziller M.J., Gu H., Müller F., Donaghey J., Tsai L.T., Kohlbacher O., De Jager P.L., Rosen E.D., Bennett D.A., Bernstein B.E. (2013). Charting a dynamic DNA methylation landscape of the human genome. Nature.

[37] Long H.K., King H.W., Patient R.K., Odom D.T., Klose R.J. (2016). Protection of CpG islands from DNA methylation is DNA-encoded and evolutionarily conserved. Nucleic Acids Res..

[38] Stadler M.B., Murr R., Burger L., Ivanek R., Lienert F., Schöler A., van Nimwegen E., Wirbelauer C., Oakeley E.J., Gaidatzis D. (2011). DNA-binding factors shape the mouse methylome at distal regulatory regions. Nature.

[39] Onuchic V., Lurie E., Carrero I., Pawliczek P., Patel R.Y., Rozowsky J., Galeev T., Huang Z., Altshuler R.C., Zhang Z. (2018). Allele-specific epigenome maps reveal sequence-dependent stochastic switching at regulatory loci. Science.

[40] Zhang Y., Rohde C., Reinhardt R., Voelcker-Rehage C., Jeltsch A. (2009). Non-imprinted allele-specific DNA methylation on human autosomes. Genome Biol..

[41] Xu Y., Wu F., Tan L., Kong L., Xiong L., Deng J., Barbera A.J., Zheng L., Zhang H., Huang S. (2011). Genome-wide regulation of 5hmC, 5mC, and gene expression by Tet1 hydroxylase in mouse embryonic stem cells. Mol. Cell.

[42] Pastor W.A., Pape U.J., Huang Y., Henderson H.R., Lister R., Ko M., McLoughlin E.M., Brudno Y., Mahapatra S., Kapranov P. (2011). Genome-wide mapping of 5-hydroxymethylcytosine in embryonic stem cells. Nature.

[43] Xu C., Liu K., Lei M., Yang A., Li Y., Hughes T.R., Min J. (2018). DNA Sequence recognition of human CXXC domains and their structural determinants. Structure.

[44] Long H.K., Blackledge N.P., Klose R.J. (2013). ZF-CxxC domain-containing proteins, CpG islands and the chromatin connection. Biochem. Soc. Trans..

[45] Tanaka Y., Umata T., Okamoto K., Obuse C., Tsuneoka M. (2014). CxxC-ZF domain is needed for KDM2A to demethylate histone in rDNA promoter in response to starvation. Cell Struct. Funct..

[46] Hashimoto H., Vertino P.M., Cheng X. (2010). Molecular coupling of DNA methylation and histone methylation. Epigenomics.

[47] Melamed P., Yosefzon Y., David C., Tsukerman A., Pnueli L. (2018). TET enzymes, variants, and differential effects on function. Front. Cell Dev. Biol..

[48] Chahrour M., Jung S.Y., Shaw C., Zhou X., Wong S.T., Qin J., Zoghbi H.Y. (2008). MeCP2, a key contributor to neurological disease, activates and represses transcription. Science.

[49] Ben-Shachar S., Chahrour M., Thaller C., Shaw C.A., Zoghbi H.Y. (2009). Mouse models of MeCP2 disorders share gene expression changes in the cerebellum and hypothalamus. Hum. Mol. Genet..

[50] Sugino K., Hempel C.M., Okaty B.W., Arnson H.A., Kato S., Dani V.S., Nelson S.B. (2014). Cell-type-specific repression by methyl-CpG-binding protein 2 is biased toward long genes. J. Neurosci..

[51] Rajavelu A., Lungu C., Emperle M., Dukatz M., Bröhm A., Broche J., Hanelt I., Parsa E., Schiffers S., Karnik R. (2018). Chromatin-dependent allosteric regulation of DNMT3A activity by MeCP2. Nucleic Acids Res..

[52] Reizel Y., Sabag O., Skversky Y., Spiro A., Steinberg B., Bernstein D., Wang A., Kieckhaefer J., Li C., Pikarsky E. (2018). Postnatal DNA demethylation and its role in tissue maturation. Nat. Commun..

[53] Yin Y., Morgunova E., Jolma A., Kaasinen E., Sahu B., Khund-Sayeed S., Das P.K., Kivioja T., Dave K., Zhong F. (2017). Impact of cytosine methylation on DNA binding specificities of human transcription factors. Science.

[54] Kribelbauer J.F., Laptenko O., Chen S., Martini G.D., Freed-Pastor W.A., Prives C., Mann R.S., Bussemaker H.J. (2017). Quantitative analysis of the dna methylation sensitivity of transcription factor complexes. Cell Rep..

[55] Maurano M.T., Wang H., John S., Shafer A., Canfield T., Lee K., Stamatoyannopoulos J.A. (2015). Role of DNA methylation in modulating transcription factor occupancy. Cell Rep..

[56] Hashimoto H., Wang D., Horton J.R., Zhang X., Corces V.G., Cheng X. (2017). Structural basis for the versatile and methylation-dependent binding of CTCF to DNA. Mol. Cell.

[57] Baubec T., Schubeler D. (2014). Genomic patterns and context specific interpretation of DNA methylation. Curr. Opin. Genet. Dev..

[58] Shimbo T., Wade P.A. (2016). Proteins that read DNA methylation. Adv. Exp. Med. Biol..

[59] Flavahan W.A., Drier Y., Liau B.B., Gillespie S.M., Venteicher A.S., Stemmer-Rachamimov A.O., Suvà M.L., Bernstein B.E. (2016). Insulator dysfunction and oncogene activation in IDH mutant gliomas. Nature.

[60] Mayran A., Khetchoumian K., Hariri F., Pastinen T., Gauthier Y., Balsalobre A., Drouin J. (2018). Pioneer factor Pax7 deploys a stable enhancer repertoire for specification of cell fate. Nat. Genet..

[61] Wan J., Su Y., Song Q., Tung B., Oyinlade O., Liu S., Ying M., Ming G.L., Song H., Qian J. (2017). Methylated cis-regulatory elements mediate KLF4-dependent gene transactivation and cell migration. Elife.

[62] Charlet J., Duymich C.E., Lay F.D., Mundbjerg K., Dalsgaard Sørensen K., Liang G., Jones P.A. (2016). Bivalent regions of cytosine methylation and H3K27 acetylation suggest an active role for DNA methylation at enhancers. Mol. Cell.

[63] Rinaldi L., Datta D., Serrat J., Morey L., Solanas G., Avgustinova A., Blanco E., Pons J.I., Matallanas D., Von Kriegsheim A. (2016). Dnmt3a and Dnmt3b associate with enhancers to regulate human epidermal stem cell homeostasis. Cell Stem Cell.

[64] Roulois D., Loo Yau H., Singhania R., Wang Y., Danesh A., Shen S.Y., Han H., Liang G., Jones P.A., Pugh T.J. (2015). DNA-demethylating agents target colorectal cancer cells by inducing viral mimicry by endogenous transcripts. Cell.

[65] Brocks D., Schmidt C.R., Daskalakis M., Jang H.S., Shah N.M., Li D., Li J., Zhang B., Hou Y., Laudato S. (2017). DNMT and HDAC inhibitors induce cryptic transcription start sites encoded in long terminal repeats. Nat. Genet..

[66] Su J., Shao X., Liu H., Liu S., Wu Q., Zhang Y. (2012). Genome-wide dynamic changes of DNA methylation of repetitive elements in human embryonic stem cells and fetal fibroblasts. Genomics.

[67] Jurkowska R.Z., Qin S., Kungulovski G., Tempel W., Liu Y., Bashtrykov P., Stiefelmaier J., Jurkowski T.P., Kudithipudi S., Weirich S. (2017). H3K14ac is linked to methylation of H3K9 by the triple Tudor domain of SETDB1. Nat. Commun..

[68] Lupo A., Cesaro E., Montano G., Zurlo D., Izzo P., Costanzo P. (2013). KRAB-Zinc finger proteins: A repressor family displaying multiple biological functions. Curr. Genomics.

[69] Fasching L., Kapopoulou A., Sachdeva R., Petri R., Jönsson M.E., Männe C., Turelli P., Jern P., Cammas F., Trono D. (2015). TRIM28 represses transcription of endogenous retroviruses in neural progenitor cells. Cell Rep..

[70] Egger G., Jeong S., Escobar S.G., Cortez C.C., Li T.W., Saito Y., Yoo C.B., Jones P.A., Liang G. (2006). Identification of DNMT1 (DNA methyltransferase 1) hypomorphs in somatic knockouts suggests an essential role for DNMT1 in cell survival. Proc. Natl. Acad. Sci. USA.

[71] Siomi M.C., Sato K., Pezic D., Aravin A.A. (2011). PIWI-interacting small RNAs: The vanguard of genome defence. Nat. Rev. Mol. Cell Biol..

[72] Xie W., Schultz M.D., Lister R., Hou Z., Rajagopal N., Ray P., Whitaker J.W., Tian S., Hawkins R.D., Leung D. (2013). Epigenomic analysis of multilineage differentiation of human embryonic stem cells. Cell.

[73] Jeong M., Sun D., Luo M., Huang Y., Challen G.A., Rodriguez B., Zhang X., Chavez L., Wang H., Hannah R. (2014). Large conserved domains of low DNA methylation maintained by Dnmt3a. Nat. Genet..

[74] Zhang Y., Jurkowska R., Soeroes S., Rajavelu A., Dhayalan A., Bock I., Rathert P., Brandt O., Reinhardt R., Fischle W. (2010). Chromatin methylation activity of Dnmt3a and Dnmt3a/3L is guided by interaction of the ADD domain with the histone H3 tail. Nucleic Acids Res..

[75] Noh K.M., Wang H., Kim H.R., Wenderski W., Fang F., Li C.H., Dewell S., Hughes S.H., Melnick A.M., Patel D.J. (2015). Engineering of a histone-recognition domain in Dnmt3a alters the epigenetic landscape and phenotypic features of mouse ESCs. Mol. Cell.

[76] Petell C.J., Alabdi L., He M., San Miguel P., Rose R., Gowher H. (2016). An epigenetic switch regulates de novo DNA methylation at a subset of pluripotency gene enhancers during embryonic stem cell differentiation. Nucleic Acids Res..

[77] Wagner E.J., Carpenter P.B. (2012). Understanding the language of Lys36 methylation at histone H3. Nat. Rev. Mol. Cell Biol..

[78] McDaniel S.L., Strahl B.D. (2017). Shaping the cellular landscape with Set2/SETD2 methylation. Cell. Mol. Life Sci..

[79] Ball M.P., Li J.B., Gao Y., Lee J.H., LeProust E.M., Park I.H., Xie B., Daley G.Q., Church G.M. (2009). Targeted and genome-scale strategies reveal gene-body methylation signatures in human cells. Nat. Biotechnol..

[80] Baubec T., Colombo D.F., Wirbelauer C., Schmidt J., Burger L., Krebs A.R., Akalin A., Schübeler D. (2015). Genomic profiling of DNA methyltransferases reveals a role for DNMT3B in genic methylation. Nature.

[81] Neri F., Rapelli S., Krepelova A., Incarnato D., Parlato C., Basile G., Maldotti M., Anselmi F., Oliviero S. (2017). Intragenic DNA methylation prevents spurious transcription initiation. Nature.

[82] Maunakea A.K., Nagarajan R.P., Bilenky M., Ballinger T.J., D’Souza C., Fouse S.D., Johnson B.E., Hong C., Nielsen C., Zhao Y. (2010). Conserved role of intragenic DNA methylation in regulating alternative promoters. Nature.

[83] Qin W., Wolf P., Liu N., Link S., Smets M., La Mastra F., Forné I., Pichler G., Hörl D., Fellinger K. (2015). DNA methylation requires a DNMT1 ubiquitin interacting motif (UIM) and histone ubiquitination. Cell Res..

[84] Yang X., Han H., De Carvalho D.D., Lay F.D., Jones P.A., Liang G. (2014). Gene body methylation can alter gene expression and is a therapeutic target in cancer. Cancer Cell.

[85] Su J., Huang Y.H., Cui X., Wang X., Zhang X., Lei Y., Xu J., Lin X., Chen K., Lv J. (2018). Homeobox oncogene activation by pan-cancer DNA hypermethylation. Genome Biol..

[86] Vire E., Brenner C., Deplus R., Blanchon L., Fraga M., Didelot C., Morey L., Van Eynde A., Bernard D., Vanderwinden J.M. (2006). The Polycomb group protein EZH2 directly controls DNA methylation. Nature.

[87] Widschwendter M., Fiegl H., Egle D., Mueller-Holzner E., Spizzo G., Marth C., Weisenberger D.J., Campan M., Young J., Jacobs I. (2007). Epigenetic stem cell signature in cancer. Nat. Genet..

[88] Schlesinger Y., Straussman R., Keshet I., Farkash S., Hecht M., Zimmerman J., Eden E., Yakhini Z., Ben-Shushan E., Reubinoff B.E. (2007). Polycomb-mediated methylation on Lys27 of histone H3 pre-marks genes for de novo methylation in cancer. Nat. Genet..

[89] Brinkman A.B., Gu H., Bartels S.J., Zhang Y., Matarese F., Simmer F., Marks H., Bock C., Gnirke A., Meissner A. (2012). Sequential ChIP-bisulfite sequencing enables direct genome-scale investigation of chromatin and DNA methylation cross-talk. Genome Res..

[90] Galupa R., Heard E. (2015). X-chromosome inactivation: New insights into cis and trans regulation. Curr. Opin. Genet. Dev..

[91] Wang X., Paucek R.D., Gooding A.R., Brown Z.Z., Ge E.J., Muir T.W., Cech T.R. (2017). Molecular analysis of PRC2 recruitment to DNA in chromatin and its inhibition by RNA. Nat. Struct. Mol. Biol..

[92] Holoch D., Margueron R. (2017). Mechanisms Regulating PRC2 Recruitment and Enzymatic Activity. Trends Biochem. Sci..

[93] Li Y., Zheng H., Wang Q., Zhou C., Wei L., Liu X., Zhang W., Zhang Y., Du Z., Wang X. (2018). Genome-wide analyses reveal a role of Polycomb in promoting hypomethylation of DNA methylation valleys. Genome Biol..

[94] Tamaru H., Selker E.U. (2001). A histone H3 methyltransferase controls DNA methylation in *Neurospora crassa*. Nature.

[95] Arand J., Spieler D., Karius T., Branco M.R., Meilinger D., Meissner A., Jenuwein T., Xu G., Leonhardt H., Wolf V. (2012). In vivo control of CpG and non-CpG DNA methylation by DNA methyltransferases. PLoS Genet..

[96] Lehnertz B., Ueda Y., Derijck A.A., Braunschweig U., Perez-Burgos L., Kubicek S., Chen T., Li E., Jenuwein T., Peters A.H. (2003). Suv39h-mediated histone H3 lysine 9 methylation directs DNA methylation to major satellite repeats at pericentric heterochromatin. Curr. Biol..

[97] Rothbart S.B., Krajewski K., Nady N., Tempel W., Xue S., Badeaux A.I., Barsyte-Lovejoy D., Martinez J.Y., Bedford M.T., Fuchs S.M. (2012). Association of UHRF1 with methylated H3K9 directs the maintenance of DNA methylation. Nat. Struct. Mol. Biol..

[98] Sharif J., Muto M., Takebayashi S., Suetake I., Iwamatsu A., Endo T.A., Shinga J., Mizutani-Koseki Y., Toyoda T., Okamura K. (2007). The SRA protein Np95 mediates epigenetic inheritance by recruiting Dnmt1 to methylated DNA. Nature.

[99] Bostick M., Kim J.K., Estève P.O., Clark A., Pradhan S., Jacobsen S.E. (2007). UHRF1 plays a role in maintaining DNA methylation in mammalian cells. Science.

[100] Berkyurek A.C., Suetake I., Arita K., Takeshita K., Nakagawa A., Shirakawa M., Tajima S. (2014). The DNA methyltransferase Dnmt1 directly interacts with the SET and RING finger-associated (SRA) domain of the multifunctional protein Uhrf1 to facilitate accession of the catalytic center to hemi-methylated DNA. J. Biol. Chem..

[101] Bashtrykov P., Rajavelu A., Hackner B., Ragozin S., Carell T., Jeltsch A. (2014). Targeted mutagenesis results in an activation of DNA methyltransferase 1 and confirms an autoinhibitory role of its RFTS domain. Chembiochem.

[102] Xie S., Jakoncic J., Qian C. (2012). UHRF1 double Tudor domain and the adjacent PHD finger act together to recognize K9me3-containing histone H3 tail. J. Mol. Biol..

[103] Rothbart S.B., Dickson B.M., Ong M.S., Krajewski K., Houliston S., Kireev D.B., Arrowsmith C.H., Strahl B.D. (2013). Multivalent histone engagement by the linked tandem Tudor and PHD domains of UHRF1 is required for the epigenetic inheritance of DNA methylation. Genes Dev..

[104] Nady N., Lemak A., Walker J.R., Avvakumov G.V., Kareta M.S., Achour M., Xue S., Duan S., Allali-Hassani A., Zuo X. (2011). Recognition of multivalent histone states associated with heterochromatin by UHRF1 protein. J. Biol. Chem..

[105] Zhao Q., Zhang J., Chen R., Wang L., Li B., Cheng H., Duan X., Zhu H., Wei W., Li J. (2016). Dissecting the precise role of H3K9 methylation in crosstalk with DNA maintenance methylation in mammals. Nat. Commun..

[106] Veland N., Hardikar S., Zhong Y., Gayatri S., Dan J., Strahl B.D., Rothbart S.B., Bedford M.T., Chen T. (2017). The arginine methyltransferase PRMT6 regulates DNA methylation and contributes to global DNA hypomethylation in cancer. Cell Rep..

[107] Jorgensen S., Schotta G., Sorensen C.S. (2013). Histone H4 lysine 20 methylation: Key player in epigenetic regulation of genomic integrity. Nucleic Acids Res..

[108] Zhu H., Geiman T.M., Xi S., Jiang Q., Schmidtmann A., Chen T., Li E., Muegge K. (2006). Lsh is involved in de novo methylation of DNA. EMBO J..

[109] Yu W., McIntosh C., Lister R., Zhu I., Han Y., Ren J., Landsman D., Lee E., Briones V., Terashima M. (2014). Genome-wide DNA methylation patterns in LSH mutant reveals de-repression of repeat elements and redundant epigenetic silencing pathways. Genome Res..

[110] Lungu C., Muegge K., Jeltsch A., Jurkowska R.Z. (2015). An ATPase-deficient variant of the SNF2 family member HELLS shows altered dynamics at pericentromeric heterochromatin. J. Mol. Biol..

[111] Ren J., Briones V., Barbour S., Yu W., Han Y., Terashima M., Muegge K. (2015). The ATP binding site of the chromatin remodeling homolog Lsh is required for nucleosome density and de novo DNA methylation at repeat sequences. Nucleic Acids Res..

[112] Song J., Teplova M., Ishibe-Murakami S., Patel D.J. (2012). Structure-based mechanistic insights into DNMT1-mediated maintenance DNA methylation. Science.

[113] Zhang Z.M., Lu R., Wang P., Yu Y., Chen D., Gao L., Liu S., Ji D., Rothbart S.B., Wang Y. (2018). Structural basis for DNMT3A-mediated de novo DNA methylation. Nature.

[114] Okuwaki M., Verreault A. (2004). Maintenance DNA methylation of nucleosome core particles. J. Biol. Chem..

[115] Gowher H., Stockdale C.J., Goyal R., Ferreira H., Owen-Hughes T., Jeltsch A. (2005). De novo methylation of nucleosomal DNA by the mammalian Dnmt1 and Dnmt3A DNA methyltransferases. Biochemistry.

[116] Takeshima H., Suetake I., Shimahara H., Ura K., Tate S., Tajima S. (2006). Distinct DNA methylation activity of Dnmt3a and Dnmt3b towards naked and nucleosomal DNA. J. Biochem..

[117] Felle M., Hoffmeister H., Rothammer J., Fuchs A., Exler J.H., Längst G. (2011). Nucleosomes protect DNA from DNA methylation in vivo and in vitro. Nucleic Acids Res..

[118] Schrader A. (2015). Characterization of Dnmt1 binding and DNA methylation on nucleosomes and nucleosomal arrays. PLoS ONE.

[119] Xu G.L., Bestor T.H., Bourc’his D., Hsieh C.L., Tommerup N., Bugge M., Hulten M., Qu X., Russo J.J., Viegas-Péquignot E. (1999). Chromosome instability and immunodeficiency syndrome caused by mutations in a DNA methyltransferase gene. Nature.

[120] Okano M., Bell D.W., Haber D.A., Li E. (1999). DNA methyltransferases Dnmt3a and Dnmt3b are essential for de novo methylation and mammalian development. Cell.

[121] De Greef J.C., Wang J., Balog J., den Dunnen J.T., Frants R.R., Straasheijm K.R., Aytekin C., van der Burg M., Duprez L., Ferster A. (2011). Mutations in ZBTB24 are associated with immunodeficiency, centromeric instability, and facial anomalies syndrome type 2. Am. J. Hum. Genet..

[122] Thijssen P.E., Ito Y., Grillo G., Wang J., Velasco G., Nitta H., Unoki M., Yoshihara M., Suyama M., Sun Y. (2015). Mutations in CDCA7 and HELLS cause immunodeficiency-centromeric instability-facial anomalies syndrome. Nat. Commun..

[123] Wu T.P., Wang T., Seetin M.G., Lai Y., Zhu S., Lin K., Liu Y., Byrum S.D., Mackintosh S.G., Zhong M. (2016). DNA methylation on *N*(6)-adenine in mammalian embryonic stem cells. Nature.

[124] Jenness C., Giunta S., Müller M.M., Kimura H., Muir T.W., Funabiki H. (2018). HELLS and CDCA7 comprise a bipartite nucleosome remodeling complex defective in ICF syndrome. Proc. Natl. Acad. Sci. USA.

[125] Bashtrykov P., Ragozin S., Jeltsch A. (2012). Mechanistic details of the DNA recognition by the Dnmt1 DNA methyltransferase. FEBS Lett..

[126] He Y., Ecker J.R. (2015). Non-CG methylation in the human genome. Annu. Rev. Genomics Hum. Genet..

[127] Lee J.H., Park S.J., Nakai K. (2017). Differential landscape of non-CpG methylation in embryonic stem cells and neurons caused by DNMT3s. Sci. Rep..

[128] Kinde B., Gabel H.W., Gilbert C.S., Griffith E.C., Greenberg M.E. (2015). Reading the unique DNA methylation landscape of the brain: Non-CpG methylation, hydroxymethylation, and MeCP2. Proc. Natl. Acad. Sci. USA.

[129] Kinde B., Wu D.Y., Greenberg M.E., Gabel H.W. (2016). DNA methylation in the gene body influences MeCP2-mediated gene repression. Proc. Natl. Acad. Sci. USA.

[130] Cheng J., Yang H., Fang J., Ma L., Gong R., Wang P., Li Z., Xu Y. (2015). Molecular mechanism for USP7-mediated DNMT1 stabilization by acetylation. Nat. Commun..

[131] Keown C.L., Berletch J.B., Castanon R., Nery J.R., Disteche C.M., Ecker J.R., Mukamel E.A. (2017). Allele-specific non-CG DNA methylation marks domains of active chromatin in female mouse brain. Proc. Natl. Acad. Sci. USA.

[132] O’Brown Z.K., Greer E.L. (2016). N6-Methyladenine: A conserved and dynamic DNA mark. Adv. Exp. Med. Biol..

[133] Greer E.L., Blanco M.A., Gu L., Sendinc E., Liu J., Aristizábal-Corrales D., Hsu C.H., Aravind L., He C., Shi Y. (2015). DNA methylation on N6-Adenine in *C. elegans*. Cell.

[134] Zhang G., Huang H., Liu D., Cheng Y., Liu X., Zhang W., Yin R., Zhang D., Zhang P., Liu J. (2015). N6-methyladenine DNA modification in *Drosophila*. Cell.

[135] Koziol M.J., Bradshaw C.R., Allen G.E., Costa A.S.H., Frezza C., Gurdon J.B. (2016). Identification of methylated deoxyadenosines in vertebrates reveals diversity in DNA modifications. Nat. Struct. Mol. Biol..

[136] Xiao C.L., Zhu S., He M., Chen D., Zhang Q., Chen Y., Yu G., Liu J., Xie S.Q., Luo F. (2018). *N*^6^-methyladenine DNA modification in the human genome. Mol. Cell.

[137] Schiffers S., Ebert C., Rahimoff R., Kosmatchev O., Steinbacher J., Bohne A.V., Spada F., Michalakis S., Nickelsen J., Müller M. (2017). Quantitative LC-MS provides no evidence for m^6^ dA or m^4^ dC in the genome of mouse embryonic stem cells and tissues. Angew. Chem. Int. Ed. Engl..

[138] Van der Wijst M.G., Rots M.G. (2015). Mitochondrial epigenetics: An overlooked layer of regulation?. Trends Genet..

[139] Saini S.K., Mangalhara K.C., Prakasam G., Bamezai R.N.K. (2017). DNA Methyltransferase1 (DNMT1) Isoform3 methylates mitochondrial genome and modulates its biology. Sci. Rep..

[140] Van der Wijst M.G., van Tilburg A.Y., Ruiters M.H., Rots M.G. (2017). Experimental mitochondria-targeted DNA methylation identifies GpC methylation, not CpG methylation, as potential regulator of mitochondrial gene expression. Sci. Rep..

[141] Liu B., Du Q., Chen L., Fu G., Li S., Fu L., Zhang X., Ma C., Bin C. (2016). CpG methylation patterns of human mitochondrial DNA. Sci. Rep..

[142] Mechta M., Ingerslev L.R., Fabre O., Picard M., Barrès R. (2017). Evidence suggesting absence of mitochondrial DNA methylation. Front. Genet..

[143] Kungulovski G., Jeltsch A. (2016). Epigenome editing: State of the art, concepts, and perspectives. Trends Genet..

[144] Rots M.G., Jeltsch A. (2018). Editing the epigenome: Overview, open questions, and directions of future development. Methods Mol. Biol..

