# Molecular Processes Connecting DNA Methylation Patterns with DNA Methyltransferases and Histone Modifications in Mammalian Genomes

**DOI:** 10.3390/genes10050388

**Published:** 2019-05-21

**Authors:** Albert Jeltsch, Julian Broche, Pavel Bashtrykov

**Affiliations:** Department of Biochemistry, Institute of Biochemistry and Technical Biochemistry, University of Stuttgart, 70569 Stuttgart, Germany; Julian.Broche@ibtb.uni-stuttgart.de (J.B.); Pavel.Bashtrykov@ibtb.uni-stuttgart.de (P.B.)

The authors wish to make the following correction to their paper [1]. An error occurred in the setup of Figure 1A: The arrows indicating the methylation and demethylation direction were incorrect and signed the inverse order of the methylation cycle. The corrected figure is shown below.

The authors would like to apologize for any inconvenience caused. The change does not affect the scientific results. The manuscript will be updated and the original will remain online on the article webpage.

## Figures and Tables

**Figure 1:**
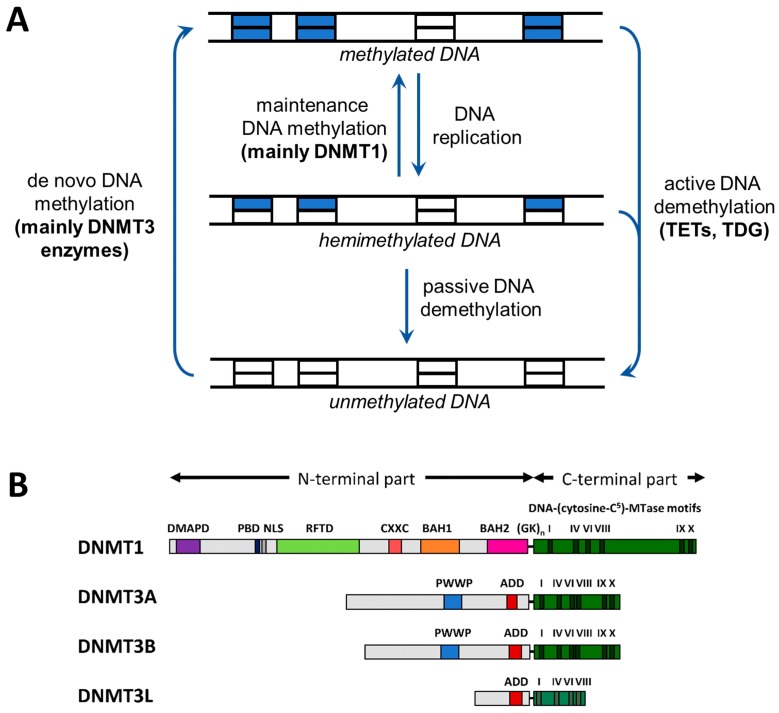
Cycle of DNA methylation and domain structure of DNA methyltransferases (DNMTs). (**A**) Cycle of DNA methylation in human cells (adapted from [9]). DNA methylation patterns are generated by de novo methyltransferases and kept through DNA replication by maintenance methylation. DNA methylation can be lost through passive or active demethylation (abbreviations: TET, Ten-eleven translocation enzyme; TDG, thymine-DNA glycosylase). (**B**) Domain structure of the mammalian DNMTs DNMT1, DNMT3A, and DNMT3B. DNMT3L is a catalytically-inactive member of the DNMT3 family, which has regulatory roles [15]. The human DNMT1, DNMT3A, DNMT3B, and DNMT3L proteins consist of 1616, 912, 853, and 387 amino acid residues, respectively. Abbreviations used: DMAPD, DNA methyltransferase-associated protein 1 interacting domain; PBD, PCNA binding domain; NLS, nuclear localization signal; RFTD, replication foci targeting domain; CXXC, CXXC domain; BAH1 and BAH2, bromo-adjacent homology domains 1 and 2; GKn, glycine lysine repeats; PWWP, PWWP domain; and ADD, ATRX-DNMT3-DNMT3L domain (reprinted from [15] with permission).
