# Supplementary material for: Molecular Processes Connecting DNA Methylation Patterns with DNA Methyltransferases and Histone Modifications in Mammalian Genomes
Source: Genes (Basel). 2018 Nov 21;9(11):566. doi: 10.3390/genes9110566 (PMC6266221; doi:10.3390/genes9110566)
Supplement: Supplementary file 1 [file genes-09-00566-s001.pdf]

Molecular processes connecting DNA methylation patterns and Histone modification in mammalian genomes

Albert Jeltsch*, Julian Broche, Pavel Bashtrykov

Supplemental Text 1

Sources of the datasets shown in Figure 4.

Modification	Series/Submission	Dataset	Sample name	Reference
H3K27me3	DRA001232	DRX013192	HEK293 H3K27me3	[1]
H3K9me3	GSE66530	GSM1624502	309M3-B lot1_ChIPSeq experiment 2	[2]
H3K79me3	GSE89052	SRX2263477	CTL H3K79me3_ChIPSeq	[3]
H4K20me3	GSE85940	GSM2288151	CL0110_H4K20me3_PAR_02 _REP2_119	[4]
POLR2A	GSE31477	GSM935534	Yale_ChipSeq_HEK293_Pol2 _std	ENCODE
H3K4me3	GSE35583	GSM945288	UW_ChipSeq_HEK293_H3K4 me3	ENCODE

References

1. Matsumoto K, Suzuki A, Wakaguri H, Sugano S, Suzuki Y. (2014) Construction of mate pair full-length cDNAs libraries and characterization of transcriptional start sites and termination sites. Nucleic Acids Res. 42(16):e125.
2. Hattori T, Lai D, Dementieva IS, Montaño SP, Kurosawa K, Zheng Y, Akin LR, Świst-Rosowska KM, Grzybowski AT, Koide A, Krajewski K, Strahl BD, Kelleher NL, Ruthenburg AJ, Koide S. (2016) Antigen clasping by two antigen-binding sites of an exceptionally specific antibody for histone methylation. Proc Natl Acad Sci U S A. 113(8):2092-7
3. Kang JY, Kim JY, Kim KB, Park JW, Cho H, Hahm JY, Chae YC, Kim D, Kook H, Rhee S, Ha NC, Seo SB. (2018) KDM2B is a histone H3K79 demethylase and induces transcriptional repression via sirtuin-1-mediated chromatin silencing. FASEB J. 32(10):5737-5750.
4. Turcan S, Makarov V, Taranda J, Wang Y, Fabius AWM, Wu W, Zheng Y, El-Amine N, Haddock S, Nanjangud G, LeKaye HC, Brennan C, Cross J, Huse JT, Kelleher NL, Osten P, Thompson CB, Chan TA. (2018) Mutant-IDH1-dependent chromatin state reprogramming, reversibility, and persistence. Nat Genet. 50(1):62-72. (Comment: data are from atrocytes. They are shown because no dataset for HEK293 or a related cell line is available.)
